# Measuring emotional preoperative stress by an app approach and its applicability to predict postoperative pain

**DOI:** 10.1371/journal.pone.0263275

**Published:** 2022-02-16

**Authors:** Carolina L. Schiavo, Rogério B. Borges, Stela M. J. Castro, Anelise S. Wolmeister, Andressa de Souza, Otávio R. S. Martins, Gabriela S. Galvão, Kahio C. K. Nazario, Fabian J. Nickel, Wolnei Caumo, Luciana C. Stefani

**Affiliations:** 1 Graduate Program in Medical Sciences, School of Medicine, Universidade Federal do Rio Grande do Sul (UFRGS), Porto Alegre, RS, Brazil; 2 Laboratory of Pain & Neuromodulation, Hospital de Clínicas de Porto Alegre (HCPA), Porto Alegre, RS, Brazil; 3 Anaesthesia and Perioperative Medicine Service, HCPA, Porto Alegre, RS, Brazil; 4 Graduate Program in Epidemiology, School of Medicine, UFRGS, Porto Alegre, RS, Brazil; 5 Department of Statistics, UFRGS, Porto Alegre, RS, Brazil; 6 School of Medicine, UFRGS, Porto Alegre, RS, Brazil; 7 Department of Surgery, School of Medicine, UFRGS, Porto Alegre, RS, Brazil; Universidad de Leon, SPAIN

## Abstract

**Background:**

The Brief Measure of Emotional Preoperative Stress (B-MEPS) was developed to evaluate the preoperative individual vulnerability to emotional stress. To obtain a refined version of B-MEPS suitable for an app approach, this study aimed: (i) to identify items with more discriminant properties; (ii) to classify the level of preoperative emotional stress based on cut-off points; (iii) to assess concurrent validity through correlation with the Central Sensitization Inventory (CSI) score; (iv) to confirm whether the refined version of B-MEPS is an adequate predictive measure for identification of patients prone to intense postoperative pain.

**Methods:**

We include 1016 patients who had undergone surgical procedures in a teaching hospital. The generalized partial credit model of item response theory and latent class model were employed, respectively, to reduce the number of items and to create cut-off points. We applied the CSI and assessed pain by Visual Analog Scale (0–10) and by the amount of postoperative morphine consumption.

**Results:**

The refined B-MEPS shows satisfactory reliability (Cronbach’s alpha 0.79). Preoperative emotional stress, according to the cut-off points, is classified into categories: low, intermediate or high stress. The refined B-MEPS exhibited a linear association with the CSI scores (r2 = 0.53, p < 0.01). Patients with higher levels of emotional stress displayed a positive association with moderate to severe pain and greater morphine consumption.

**Conclusion:**

The refined version of B-MEPS, along with an interface of easy applicability, assess emotional vulnerability at the bedside before surgery. This app may support studies focused on intervening with perioperative stress levels.

## Introduction

Major surgical procedures have a huge impact on patient’s life. Perioperative professionals are responsible for improving patient’s perioperative experience. To achieve this goal, we should consider vulnerabilities in at least three dimensions: physical, psychological, and social [1]. Strategies have been implemented to address these dimensions. Several instruments are currently used to predict complications and even postoperative death probability using comorbidities and surgical predictors [2, 3]. In the last few years, the understanding of the surgical impact on patients’ life has improved, with a search for the best clinical results, preventing complications and promoting pre-habilitation.

On the other hand, research is still needed to understand psychological vulnerability and its impact on perioperative outcomes. Psychological predictors such as anxiety, depression [4, 5] or catastrophism [6], have been included in several models to predict postoperative pain. However, preoperative assessment rarely includes evaluation of a myriad of emotional reactions, ranging from fear of scarring and of anaesthesia, concerns about family support and, ultimately, fear of the future and death.

The brief measure of emotional preoperative stress (B-MEPS) [7] was developed to evaluate the preoperative individual vulnerability to emotional stress. The item response theory (IRT) strategy was successfully used to group together significant items from different tools currently employed to measure depressive symptoms, anxiety, minor psychiatric problems, and future self-perceptions. The B-MEPS index was an independent factor associated with moderate to intense acute postoperative pain in patients who undergo major surgery.

Although preliminary data supported the B-MEPS instrument as a valid tool, further analyses are needed to confirm the instrument’s accuracy and usefulness. Our main objective is to consolidate a preoperative measure of individual psychological vulnerability to be applied in future interventions studies. For this, we re-evaluated the psychometric properties of the first version of B-MEPS and identified cut-off values likely be used with an app to facilitate the user’s interface. As secondaries objectives, we compared the results of the B-MEPS with an established measure of central sensitization and assessed the relationship between B-MEPS and postoperative pain in a new sample of major surgeries.

## Material and methods

### Study cohorts and data collection procedures

After approval by the local research ethics committee of Hospital de Clínicas de Porto Alegre (Application 1700900), we conducted a study to validate the B-MEPS instrument from two cohorts.

The same cohort recruited for B-MEPS development was used for a retrospective analysis of the basic properties of the instrument. Data collection took place between March 2009 and March 2010. All patients aged between 18 and 70 years, ASA 2 and 3, admitted to a tertiary hospital one day before the procedure and with a minimum period of three days of hospitalization, were eligible.

From March 2017 to March 2018, we conducted a prospective observational study. One hundred fifty-three patients were sequentially recruited and followed until hospital discharge.

In each cohort, the exclusion criteria were clinical history of brain injury, history of intellectual disability or inability to cooperate and personal refusal. All patients underwent a variety of elective surgeries classified as major, considering criteria such as blood loss, grade of pain and exposure of a body cavity [8]. All participants gave their written informed consent prior to participating in the study [7].

**Fig 1:**
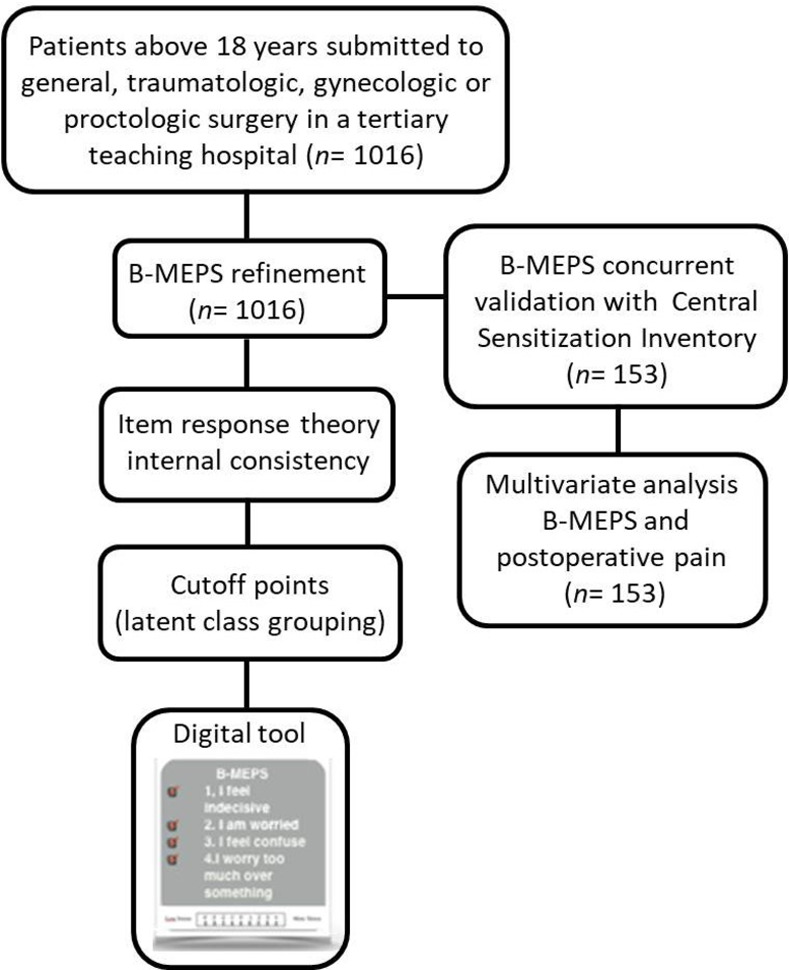
Flowchart of the study. Flowchart illustrating the selection of patients included in the study. B-MEPS: Brief Measure of Emotional Preoperative Stress.

A total of one thousand and sixteen patients were enlisted from the surgical general, proctologic, traumatology and gynaecologic units of the Hospital de Clínicas de Porto Alegre, Rio Grande do Sul. Fig 1 shows the study flowchart.

### The B-MEPS refinement

The B-MEPS is an instrument developed recently by our research group based on IRT, which is a modern psychometric approach that considers both the instrument as a whole and its individual items [7]. To develop the B-MEPS the group selected items from the following psychological instruments: the reduced version of the State-Trait Anxiety Inventory (STAI), the Montgomery and Asberg Depression Rating Scale (MADRS), the World Health Organization’s (WHO) Self-reporting Questionnaire (SRQ-20) (used to measure minor psychiatric disorders), and the Future Self-perception Questionnaire (FSPQ). Together, the questionnaires have 65 items. Afterwards, reliability was maximized by removing the least reliable questions, as indicated by the increase in Cronbach’s alpha [if any]. This procedure resulted in a final version of 15 items. We re-examined the basic properties of B-MEPS instrument using IRT and confirmed the one-dimensionality of the latent trait, the item characteristic curve, and the item discrimination related to stress level.

### Cut-off point

The IRT psychometric model conceptualizes the measurement scale like a ruler, where gradations are called items, located along the measurement scale according to their stress level. Greater accuracy was observed when stress level was roughly between one and two standard deviations from the mean [9]. To identify the level range to classify patients as more stressed in a practical way, it is necessary to establish cut-off points in the scale. This was done with the Latent Class Analysis [10], which is a statistical method that identifies distinct groups (latent classes) based on the patterns of responses observed in categorical variables.

### Concurrent validity with central sensitization inventory

Concurrent validity indicates the extent to which the result of the instrument under study correlates with another instrument that measures similar construct [11]. For this assessment, we used the Central Sensitization Inventory (CSI). CSI is a self-report questionnaire of chronic pain related to the central sensitization health symptoms that develop in response to the amplification of nociceptive inputs, in vulnerable chronic pain patients. Usually, psychological vulnerability and catastrophism are associated with central sensitization [12]. These constructs conceptually related but distinct; therefore, a moderate correlation coefficient was expected between these instruments.

### B-MEPS and postoperative pain

The possible interference of preoperative stress level in acute postoperative pain was evaluated in a subsample of 153 patients. All patients were submitted to major surgery, most of them with combined anaesthesia, with neuroaxis morphine as basal analgesia. Dipyrone and nonsteroidal anti-inflammatory drugs (NSAIDs) were given if no contra-indication was present. Postoperative analgesia was carried out with multimodal analgesia, and morphine as demanded if the pain was still not under control. Pain at rest or movement was assessed by visual analogue scale from zero (absence of pain) to 10 (worst possible pain) 12, 24 and 48 hours into the postoperative period. Additionally, morphine consumption was evaluated within the first 48 hours.

### Statistical analysis

Demographic data are presented as mean ± standard deviation (SD), median (interquartile range), number (%) or 95% confidence interval (CI). The internal consistency of the B-MEPS was assessed by Cronbach’s alpha. Item correlation was evaluated and the items that, upon deletion, contributed to increase Cronbachs’s alpha were considered for elimination. Furthermore, the refinement of the B-MEPS instrument was performed using the Generalized Partial Credit Model (GPCM) of the IRT, which is a unidimensional model of IRT used for polytomous data (items with more than two response categories) [13]. The software used for this analysis was the Latent Trait Model under IRT, R-package 1.1–1. Discrimination refers to the importance of each item for the estimation of the latent trait. This is an important component of the amount of information each item provides to estimate the preoperative stress level. Items with discrimination below 0.7 that also reduced the internal consistency (Cronbach’s alpha) were eliminated. The item information curve and the test information curve were generated to analyse the performance of the B-MEPS and to identify the interval in the latent trait the scale in which had greater precision. In general, a sample size of 500 patients provides accurate parameter estimates in IRT [14].

The latent class model was used to investigate the presence of response patterns on B-MEPS items and to establish cut-off points on the scale. The choice of the number of classes was made based on the authors’ suggestions / procedure [10, 15, 16]. To obtain the cut-off points of the B-MEPS score, we used the extended Youden index [17] with latent classes as the reference standard. Analyses were performed using the poLCA [18] and DiagTest3Grp [19] packages. From these definitions, we were able to identify, in a more practical manner, the level at which the patients might be considered more stressed.

The second part of the validation process was to examine the concurrent validity of the B-MEPS, by measuring the strength of the relationship between the B-MEPS and the Central Sensitivity Questionnaire [12]. This analysis was performed on a subsample of 153 patients, which was the sample size required for a statistical power above 99% in the correlation significance test, considering a 5% significance level. Associations were measured using Pearson’s (ρ) correlation coefficients.

Finally, the impact of preoperative stress in postoperative pain was evaluated, considering the cut-off points of B-MEPS. An ANCOVA analysis was performed to verify differences in resting and movement pain 12, 24 and 48 hours after surgery, including the covariate variables which might interfere in postoperative pain measurement, or those that exhibited statistical significance in the univariate analysis. Morphine consumption difference between high- or low- stressed patients were analysed with generalized linear model. For the B-MEPS refinement, the R statistic software, version 3.2.3, was employed. For correlation and regression analysis the SPSS version 22.0 was used.

## Results

### B-MEPS refinement

Reliability of the B-MEPS was analysed by estimating the internal consistency through the Cronbach’s alpha [20]. Three items reduced the instrument’s internal consistency. Item 11: “When I leave the hospital my life will be…”, item 12: “How do you react when you are unhappy?” Item 13: “I think about my future with…”. Excluding those items led to an increase in Cronbach’s alpha to 0.79. To confirm that some items did not contribute to the final construct, we evaluated those items’ discrimination with GPCM of the IRT [21]. The most discriminating items referred to emotional state or feelings. Item 4: “I feel confused”; Item 2: “I feel indecisive”; and the Item 9: “Do you feel unhappy?”. The items 11,12 and 13 had the lowest discrimination index (below 0.7) and were eliminated from the final version, increasing the instrument’s reliability. Table 1 shows the item correlation and Cronbach’s alpha with deleted variables. The final version of B-MEPS is displayed in the S1 Table, and the item parameters discrimination in the S2 Table.

**Table 1:** Descriptive statistics for B-MEPS items (*n* = 1016).

Item		Item total correlation	Alpha with deleted variables	Discrimination item [EP]
**1.**	I am jittery.	0.466807	0.777262	1.046 (0.100)
**2.**	I feel indecisive.	0.489678	0.774965	1.509 (0.161)
**3.**	I am worried.	0.462408	0.777033	1.074 (0.095)
**4.**	I feel confused.	0.529111	0.772343	1.774 (0.177)
**5.**	I feel like a failure.	0.347266	0.785971	0.703 (0.073)
**6.**	I worry too much over something that really does not matter.	0.475575	0.775642	1.025 (0.093)
**7.**	I take disappointments so personally that I cannot get them out of my mind.	0.484530	0.775811	1.031 (0.093)
**8.**	I get in a state of tension or turmoil as I think over my recent concerns and interests.	0.448653	0.778271	1.100 (0.098)
**9.**	Do you feel unhappy?	0.401045	0.780995	1.424 (0.165)
**10.**	Do you have feelings of discomfort in the stomach?	0.231772	0.794695	0.561 (0.091)
**11.**	When I leave the hospital my life will be	0.306747	0.787619	0.414 (0.067)
**12.**	How do you react when you are unhappy?	0.315488	0.787820	0.345 (0.057)
**13.**	I think about my future with	0.198254	0.796097	0.207 (0.053)
**14.**	How do you react when you are unhappy?	0.399525	0.780832	1.000 (0.101)
**15.**	How do you describe your depressed mood?	0.395197	0.781327	1.195 (0.135)

B-MEPS: Brief Measure of Emotional Preoperative Stress. *Total Cronbach Alpha after exclusion of items 11*,*12*,*13 was 0*,*79*.

**Fig 2:**
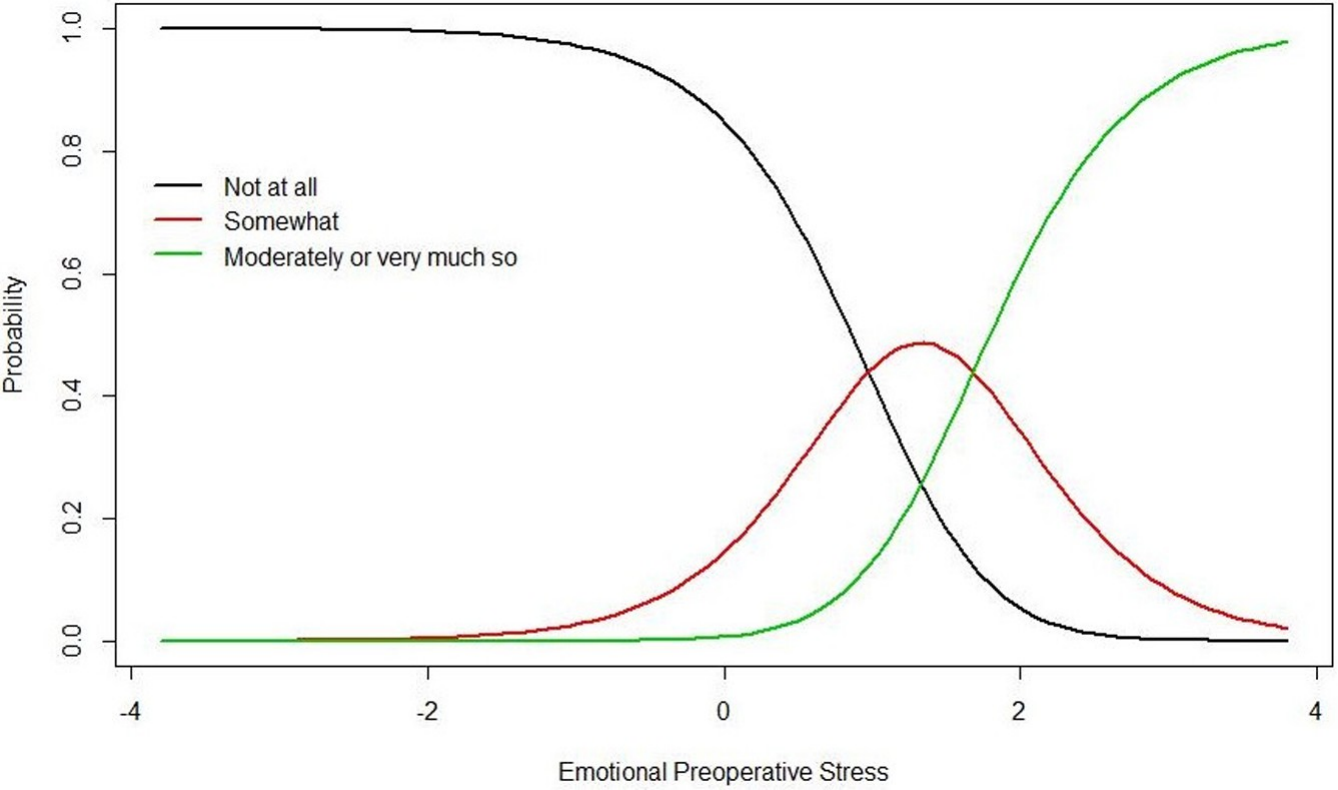
Item characteristic curve for the item B-MEPS 4: “I feel confused”. The curve shows the probability of endorsing a particular item response. The level of preoperative stress required to respond to the category “not to all” and to respond “somewhat” is 0.972 deviations above average. To change to category “moderately or very much so”, the individual needs to present a stress level of 1.681 above average. B-MEPS 4: Brief Measure of Emotional Preoperative Stress 4.

The dimensionality of the new version of the B-MEPS scale was evaluated through a Principal Component Analysis, based on the polychoric correlation matrix (used for analysis of categorical variables). A preponderant factor was found that explained 41.58% of the total variance, confirming the hypothesis of satisfactory. The unidimensional latent trait is usually evaluated through the subject’s responses to a set of items. The association between the respondent’s latent trait level and the probability of response to a particular item category is illustrated by the Item Response Category Curves. Fig 2 shows the plot of the item characteristic curves for the three categories in the scale for B-MEPS question 4. In the final version of B-MEPS instrument, the question 4 was the most discriminating item for emotional stress. A person with elevated level of preoperative stress has a higher probability of choosing the category “moderately or very much so” over the category “not at all” when responding to this question.

**Fig 3:**
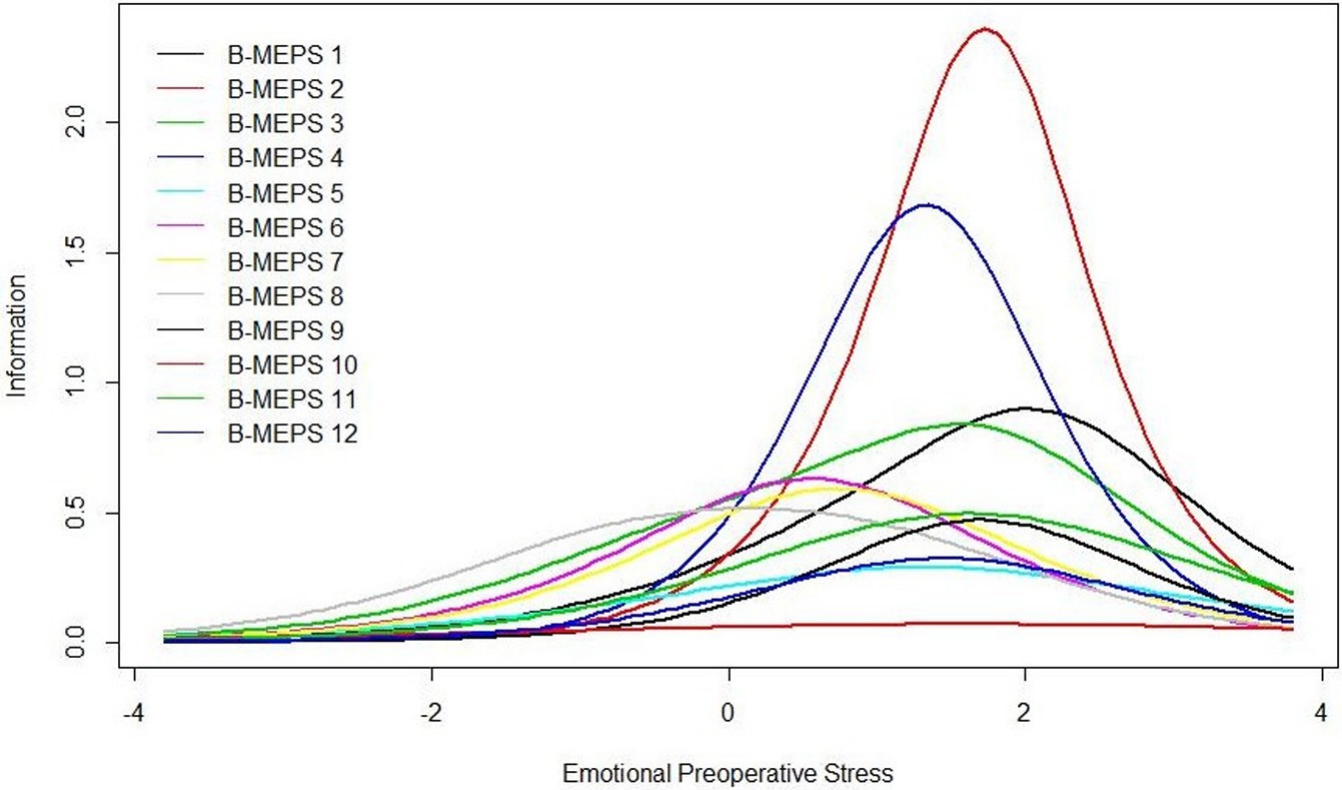
Item information curves of B-MEPS. The horizontal axis reflects the latent trait level of the test taker and the vertical axis reflects the probability of endorsing an item. The curve shows the region where the group of items (B-MEPS) estimates the latent trait of “emotional preoperative stress” with greater accuracy. The item characteristic curve represents the probability of patients positively answering the items according to their emotional stress on a continuum. B-MEPS: Brief Measure of Emotional Preoperative Stress.

The psychometric model of the IRT conceptualizes the measurement scale as a ruler, allowing for a continuous representation of preoperative stress. Fig 3 shows the Item Information Curve for each of 12 items. These curves illustrate the latent trait region in which each item contributes with more information. Even though item 4 is the most discriminative, it is not the most informative. Item 3 has the highest informative capacity. Items provide more information for estimation of the latent trait for those subjects who show a higher intensity of preoperative stress, ranging from 0.5 to 2.5 SD above average. Thus, the B-MEPS is a useful discriminatory tool, showing greater accuracy in patients who present intermediate to high levels of stress.

Aiming at a greater applicability of the tool, cut-off points were identified, categorize patients according to the intensity of preoperative psychological stress. For this, we used latent class model grouping [10]. With the new version of the B-MEPS scale, patients can be classified as having low stress (up to 0.22 SD above average), intermediate stress (between 0.22–0.77 SD) or high stress (above 0.77 SD).

**Fig 4:**
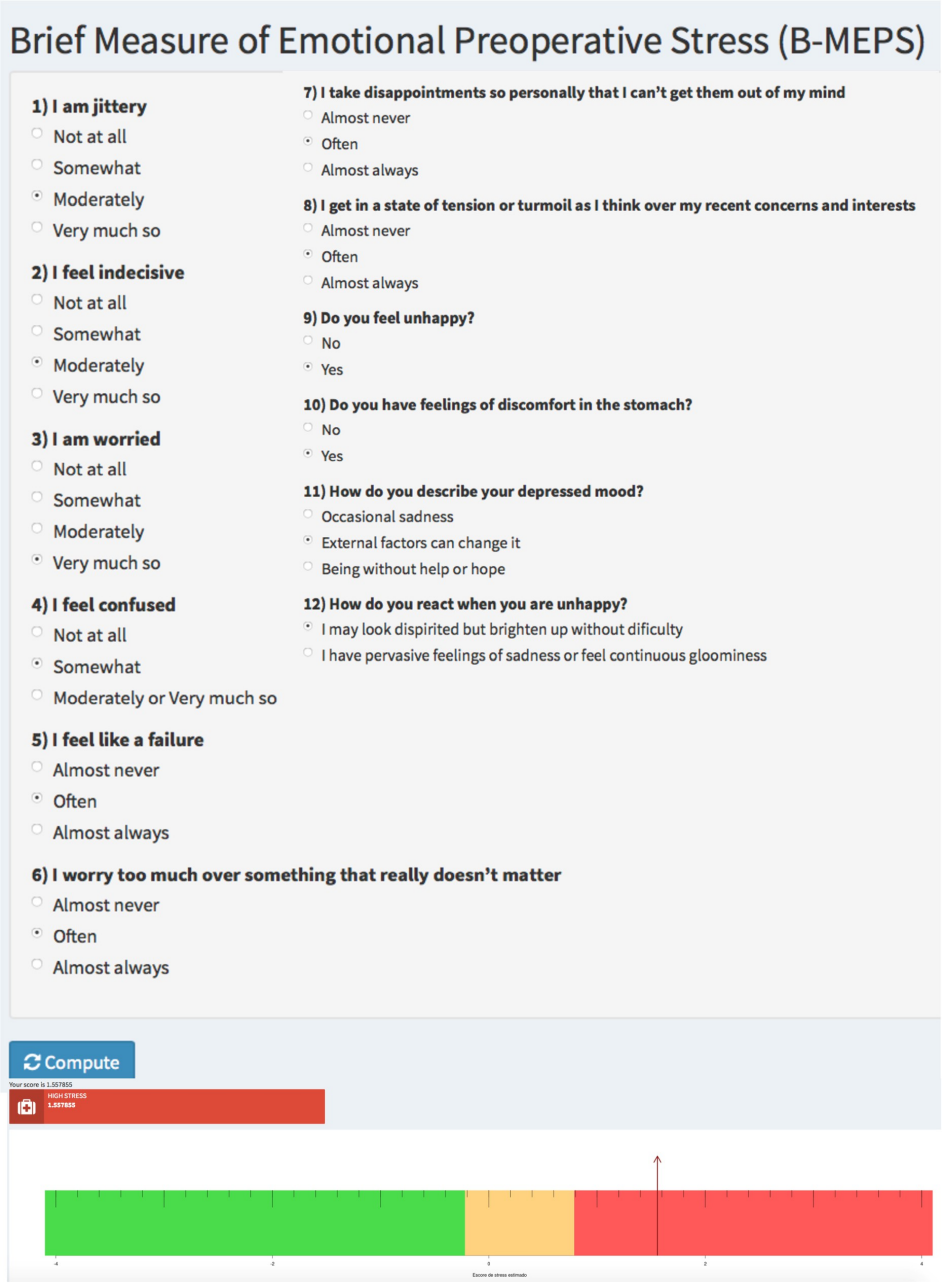
Digital tool to perform the preoperative stress calculation.

An interface for practical use and bedside application was developed using Shiny applications, a package in RStudio. This tool will be used to overcome what would otherwise be a considerable challenge: performing preoperative stress calculations based on an IRT statistic at patient’s bedside. The tool is available at https://rogerio.shinyapps.io/r_shiny/ and can be accessed for research and clinical practice purposes (Fig 4).

**Fig 5:**
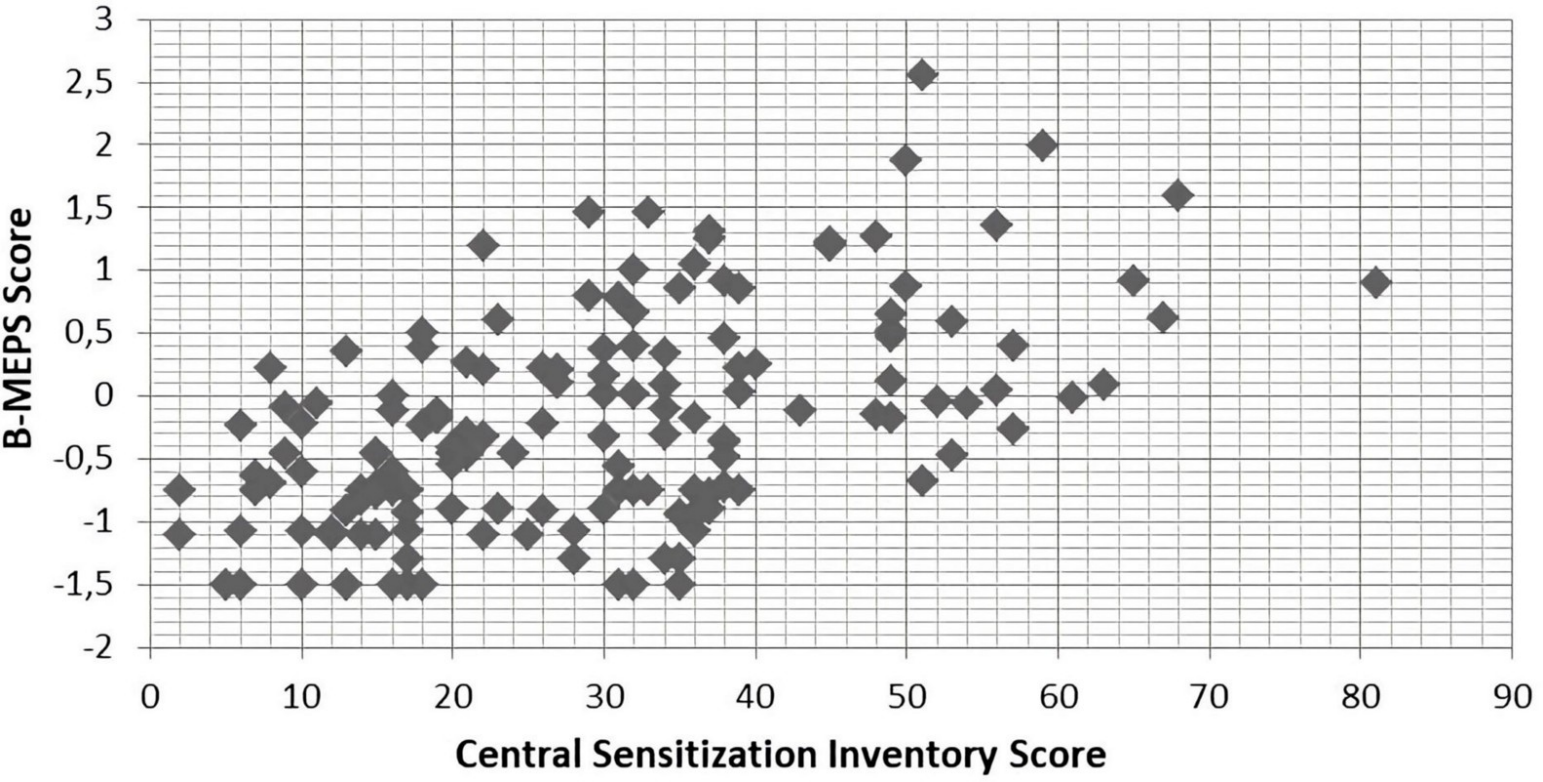
Scatter plot of B-MEPS result and Central Sensitivity Index (n = 153). B-MEPS: Brief Measure of Emotional Preoperative Stress.

The criteria validity of the B-MEPS was further established by examining the pattern of correlations between result on the B-MEPS and scores on the Central Sensitization Inventory. A moderate linear association between both scales was found (Pearson correlations 0.53, p < 0.01) indicating that similar constructs are evaluated by the two instruments. A scatterplot is shown in Fig 5.

A subsample of 153 patients who underwent to major surgery in 2017–2018 was selected and regardless of the specific kind, all have potential for moderate to severe postoperative pain. We categorized patients in two groups: one with low and intermediate preoperative stress, defined by the latent class analysis, and the other with high perioperative stress. The goal of this division between groups was to explore the association between stress level, socio-demographic factors and outcomes related to pain (Table 2). Patients with higher levels of stress exhibited a positive association with moderate to severe movement pain (VAS > 4) in 24h, even inclusion of covariates in the ANCOVA model such as sex, age, neuroaxial morphine, regional anesthesia, cancer, psychiatric diagnosis and previous pain medication use. Additionally, using a general linear model with distribution, we found higher morphine consumption (6 mg or more) at 48h in the high-group, controlling for the same variables (Table 3).

**Table 2:** General health and surgical characteristics in low or high preoperative stress patients (*n* = 153).

	Low stress (*n* = 130)		High stress (*n* = 23)		*p*
	*n*	%	*n*	%	
Age	58.35	12.47	57.7	10.55	0.81
Sex (fem)	73	56.20	14	60.90	0.67
Tabagism	14	10.80	2	8.70	0.76
Alcohol intake	14	10.80	4	17.40	0.36
Chronic pain	25	19.20	8	34.80	0.09
Chronic pain medication	26	20.00	7	30.40	0.26
Psychiatric diagnosis	22	17.00	9	39.00	0.015
Depression	16	12.30	6	26.10	0.08
Anxiety	9	12.30	5	21.70	0.02
Cancer diagnosis	65	12.30	12	52.20	0.84
General anesthesia	12	12.30	4	18.20	0.20
Morphine neuroaxial	107	12.30	14	66.70	0.08
Surgery					
Hysterectomy	23	17.80	4	19.00	0.32
Prostatectomy	15	11.60	2	9.50	0.76
Hip prothesis	15	11.60	1	4.80	0.37
Knee prothesis	19	14.70	3	14.30	0.92
Retosigmoidectomy	57	44.20	11	52.40	0.48

**Table 3:** Pain evaluation in 12, 24 and 48 hours comparing patients with high preoperative stress versus intermediate-low stress group (low stress).

**Dependent variable**	**Low stress (SE)**	**High stress (SE)**	**Mean difference (SE)**	**F**	***p***
**Pain Visual Analogue Scale**					
Rest pain 12h	3.39 (0.42)	4.10 (0.65)	-0.76 (0.71)	1*	0.31
Movement pain 12h	5.98 (0.45)	6.89 (0.68)	-0.90 (0.73)	1.52*	0.21
Rest pain 24h	3.45 (0.46)	4.31 (0.70)	-1.06 (0.76)	1.27*	0.26
Movement pain 24h	6.10 (0.41)	7.68 (0.62)	-1.48 (0.67)	4.84*	0.02
Movement pain 48h	6.26 (0.42)	6.36 (0.64)	-0.09 (0.69)	0.02*	0.88
Rest pain 48h	3.12 (0.41)	3.04 (0.63)	-0.18 (0.69)	0.01*	0.91
**Dependent variable**	**Low stress (SE)**	**High stress (SE)**	**Mean difference (SE)**	**Wald Chi-Square**	***p***
**Morphine consumption [mg]**					
Morphine consumption 12h	4.20 (0.66)	6.33 (0.91)	-2.13 (0.93)	6.45 ^†^	0.02
Morphine consumption 24h	4.26 (0.68)	5.25 (0.95)	-0.99 (0.96)	1.05 ^†^	0.3
Morphine consumption 48h	2.29 (0.59)	2.45 (0.82)	-0.80 (0.84)	0.04 ^†^	0.78

*Ancova was used for normal distribution data (rest and movement pain) and

† Multivariate analysis (general linear model) for morphine consumption; both controling for sex, age, neuroaxial morphine, regional anesthesia, cancer, psychiatric diagnosis and previous pain medication use.

## Discussion

Recently, the emerging concept of patient-centred care and the expansion of recovery to a multidimensional construct has inspired the development of tools to assess patient psychological profile before surgery and its impact on short and long-term outcomes [22]. We developed a reliable tool to access and categorize the emotional pre-operative stress through the B-MEPS index. Furthermore, we found an association between emotional pre-operative stress and central sensitization and postoperative pain.

The main contribution of our study is that we strengthened the framework of perioperative stress measured by the B-MEPS index, which is an instrument constructed with powerful IRT analysis. We refined the first version of B-MEPS, reanalysing the items in a new sample. Three items from the original version that showed low discrimination and reduced the internal consistency were excluded. The new twelve-items version confirmed the one-dimensionality of the instrument; only one latent trait is measured by the items in the scale. Also, this new version maintained the predictive capacity of identifying people with elevated preoperative stress, with higher accuracy when compared to the less stressed ones. These results indicate that the content validity of the B-MEPS index is satisfactory. Additionally, convergent validity was demonstrated by the moderate correlation between the B-MEPS and the Central Sensitivity Index, indicating that the scales measure related constructs. Central sensitization represents increased activity of nociceptive pathways circuits, caused by increased neuronal excitability and synaptic efficacy, as well as by the reduction of nociceptive inhibitory pathway activity [23, 24]. This phenomenon is responsible for alterations in pain sensitivity thresholds in acute and chronic pain situations. Therefore, we may conclude that sensitized patients have higher psychological vulnerability and are more susceptible to preoperative emotional stress.

The second finding is the establishment of cut-off points in the B-MEPS scale, using the latent class grouping. This analysis allowed us to classify patient into low, intermediate, or high emotional preoperative stress groups. This qualitative classification is important to assist in decision-making contexts. A rating system capable of measuring emotional preoperative stress level individually would benefit scenarios with limited human, financial and infrastructure resources. The team would be able to manage patients ‘stress levels, focusing on the most vulnerable- those with the highest levels of emotional stress. From this, we have developed a digital tool (for research and clinical purposes) that shares an interface with the statistical program, overcoming what would otherwise be a considerable challenge: performing a calculation based on an IRT at the patient’s bedside.

The third finding is the impact of high emotional stress on acute postoperative pain. This outcome is more difficult to evaluate and is influenced by many variables related to physical, surgical, social, and emotional variables. Nevertheless, our study confirmed the finding of higher movement pain at 24h and the morphine consumption at 12h postoperatively for more stressed patients, even after controlling for major confounds.

Further publication of our research group addressed the definition of stress related to surgery within a theoretical model focused on allostatic load [7, 24]. Three independent systems are responsible for the preservation of homeostasis when an injury occurs: endocrine, neural and immune [25]. According to this theory, stress result from psychological or physiological changes that occur when environmental stressors exceed and dysregulate an individual’s adaptive capacity (allostasis). This homeostatic disruption affects the course of chronic [26] and acute diseases as much as postoperative prognosis. A prospective study with 952 patients who underwent total knee arthroplasty showed that psychological distress (assessed by the SF-36 Quality of Life Questionnaire: Medical Outcomes Questionnaire Study) is a predictor of greater intensity of acute pain and worse postoperative functional recovery [27]. According to Kaunisto et al. [28], in breast cancer surgeries, higher levels of preoperative anxiety are related to increased perception and sensitivity to pain, and elevated postoperative acute pain scores. Patients with higher levels of anxiety are also at increased risk of developing and maintaining depressive symptoms in the late postoperative period of elective hysterectomies [29].

Therefore, it is of sum importance to include the influence of psychological factors in the perioperative research agenda as well as strategies to improve the emotional perioperative burden. Also, pain scales with multiple domains such as cognitive, affective, and sensory should be applied to better evaluate this symptom [30]. Simple interventions, such as relationship improvement and patient education with preoperative visits or group approaches [31], have already proven effective [32]. Lee and Gin [31] found that orientation meetings in small groups conducted by multidisciplinary health professionals reduced preoperative anxiety in patients undergoing total hip arthroplasty. Relaxation techniques such as mindfulness, music therapy and biofeedback have also been applied in the perioperative context aiming to reduce stress [33]. Intrahospital psychological intervention in surgical patients may help to understand individual behaviours and reactions, aiming to adapt the patient to the reality of the disease by enabling them to develop a more adequate emotional adaptation, leading to improved coping and reduced stress levels [34]. There are also evidence that preoperative or postoperative psychological interventions may reduce acute and chronic postoperative pain. The Toronto General Hospital implemented a Multidisciplinary Program to Prevent Chronic Postsurgical Pain. This program is focused on early identification of patients at risk for chronic pain after surgery, offering coordinated and comprehensive care by the multidisciplinary team consisting of pain physicians, advanced practice nurses, psychologists, and physiotherapists. The program allows to impact patients’ pain trajectories, preventing the transition from acute to chronic pain, and reducing suffering, disability, and health care costs [35].

We consider as limitations of the present study the wide variety of neuropsychological implications involved in the complex and multidimensional construct of emotional stress, along with the lack of evaluation of long-term outcomes, especially those related to the quality of rehabilitation and chronification of postoperative pain. Also, the restricted sample of prospective evaluation of acute postoperative pain, which have many confounders and variables, leads to multivariate model overfitting.

The ability of patient to deal with their surgical experience, especially in major surgeries, involves both physical and psychological aspects. The concept of Risk Management should be amplified. Stress, which is one psychological factor commonly associated with perioperative adverse events and thus negative postoperative outcomes, should be routinely assessed, including the long-term outcomes related to it. When developing B-MEPS, we intended to instrumentalize the perioperative professionals and to encourage a broader approach towards a framework for measuring emotional preoperative stress. Some strategies need to be recognized as leading priorities for subsequent research, such as patient’s education, psychological preparation programs, management of chronic preoperative pain, and individualized psychological approach.

## Conclusion

Our results confirmed that the refined version of B-MEPS is a consistent method for screening preoperative emotional stress and valuable to detect individuals prone to moderate to severe postoperative pain.

The easy-to-use app assesses emotional vulnerability at the bedside before surgery and may help the team manage patients “stress levels”, focusing on those with the highest levels of emotional stress. This app may support future studies focused on intervening with perioperative stress levels.

## Supporting information

S1 Table The final version of B-MEPS instrument. Instruction to patients: “These questions aim to assess your feelings of stress related to the perioperative period”.
(DOCX)

S2 Table Item parameters discrimination of the new B-MEPS 12 item version. (DOCX)

S3 Table STROBE statement—checklist of items that should be included in reports of observational studies. (DOCX)

S1 Data (XLSX)
