# Supplementary material for: Measuring emotional preoperative stress by an app approach and its applicability to predict postoperative pain
Source: PLoS One. 2022 Feb 16;17(2):e0263275. doi: 10.1371/journal.pone.0263275 (PMC8849448; doi:10.1371/journal.pone.0263275)
Supplement: S1 Table — Instruction to patients: “These questions aim to assess your feelings of stress related to the perioperative period”. (DOCX) [file pone.0263275.s001.docx]

**S1 Table.** The final version of B-MEPS instrument. Instruction to patients: “These questions aim to assess your feelings of stress related to the perioperative period”.

|  | **Item content** | **Response scale** | | | |
| --- | --- | --- | --- | --- | --- |
| **1.** | I am jittery | (1) not at all | (2) somewhat | (3) moderately | (4) very much so |
| **2.** | I feel indecisive | (1) not at all | (2) somewhat | (3) moderately | (4) very much so |
| **3.** | I am worried | (1) not at all | (2) somewhat | (3) moderately | (4) very much so |
| **4.** | I feel confused | (1) not at all | (2) somewhat | (3) moderately or very much so |  |
| **5.** | I feel like a failure | (1) almost never | (2) often | (3) almost always |  |
| **6.** | I worry too much over something that really does not matter | (1) almost never | (2) often | (3) almost always | |
| **7.** | I take disappointments so personally that I cannot get them out of my mind | (1) almost never | (2) often | (3) almost always | |
| **8.** | I get in a state of tension or turmoil as I think over my recent concerns and interests | (1) almost never | (2) often | (3) almost always | |
| **9.** | Do you feel unhappy? | (1) No | (2) Yes |  |  |
| **10.** | Do you have feelings of discomfort in the stomach? | (1) No | (2) Yes |  |  |
| **11.** | How do you react when you are unhappy? | (1) I may look dispirited but brighten up without difficulty | | | |
|  |  | (2) I have pervasive feelings of sadness or feel continuous gloominess | | | |
| **12.** | How do you describe your depressed mood? | (1) Ocasional sadness | | | |
|  |  | (2) External factors can change it | | | |
|  |  | (3) Being without help or hope | | | |

B-MEPS: Brief Measure of Emotional Preoperative Stress.
