# Supplementary material for: Measuring emotional preoperative stress by an app approach and its applicability to predict postoperative pain
Source: PLoS One. 2022 Feb 16;17(2):e0263275. doi: 10.1371/journal.pone.0263275 (PMC8849448; doi:10.1371/journal.pone.0263275)
Supplement: S2 Table — (DOCX) [file pone.0263275.s002.docx]

**S2 Table.** Item parameters discrimination of the new B-MEPS 12 item version.

| **Item content** | | **Discrimination (SE)** | ***b*_1,2_ (SE)** | ***b*_3,4_ (SE)** | ***b*_5,6_ (SE)** |  |
| --- | --- | --- | --- | --- | --- | --- |
| **1.** | I am jittery. | 1.021 (0.098) | 0.722 (0.101) | 2.184 (0.178) | 2.371 (0.257) | |
| **2.** | I feel indecisive. | 1.578 (0.170) | 1.235 (0.111) | 1.782 (0.128) | 2.025 (0.178) | |
| **3.** | I am worried. | 1.113 (0.098) | -0.350 (0.077) | 1.461 (0.119) | 2.144 (0.176) | |
| **4.** | I feel confused. | 1.811 (0.182) | 0.972 (0.083) | 1.681 (0.101) | - | |
| **5.** | I feel like a failure. | 0.718 (0.075) | 0.680 (0.129) | 1.931 (0.190) | - | |
| **6.** | I worry too much over something that really does not matter. | 1.049 (0.096) | 0.167 (0.088) | 0.938 (0.097) | - | |
| **7.** | I take disappointments so personally that I cannot get them out of my mind. | 1.031 (0.094) | 0.277 (0.089) | 1.183 (0.107) | - | |
| **8.** | I get in a state of tension or turmoil as I think over my recent concerns and interests. | 1.131 (0.101) | -0.835 (0.086) | 1.088 (0.094) | - | |
| **9.** | Do you feel unhappy? | 1.373 (0.162) | 1.697 (0.150) | - | - | |
| **10.** | Do you have feelings of discomfort in the stomach? | 0.526 (0.090) | 1.590 (0.285) | - | - | |
| **11.** | How do you react when you are unhappy? | 1.137 (0.131) | 1.451 (0.143) | - | - | |
| **12.** | How do you describe your depressed mood? | 1.016 (0.103) | 0.861 (0.112) | 2.389 (0.201) | - | |

B-MEPS: Brief Measure of Emotional Preoperative Stress. SE: Standard error. *b*_1,2_**_:_** Point of intersection between categories 1 and 2 (categories of answers ordered). *b*_2,3_: Point of intersection between categories 2 and 3. *b*_3,4_: Point of intersection between categories 3 and 4.
