# Supplementary material for: Measuring emotional preoperative stress by an app approach and its applicability to predict postoperative pain
Source: PLoS One. 2022 Feb 16;17(2):e0263275. doi: 10.1371/journal.pone.0263275 (PMC8849448; doi:10.1371/journal.pone.0263275)
Supplement: S3 Table — (DOCX) [file pone.0263275.s003.docx]

**S3 Table. STROBE Statement - checklist of items that should be included in reports of observational studies.**

| **Item** | **Item number** | **Recommendation** | **Pages** |
| --- | --- | --- | --- |
| **Title and abstract** | 1 | (*a*) Indicate the study’s design with a commonly used term in the title or the abstract. | 2 |
|  |  | (*b*) Provide in the abstract an informative and balanced summary of what was done and what was found. | 2 |
| **Introduction** | | |  |
| Background/rationale | 2 | Explain the scientific background and rationale for the investigation being reported. | 3 |
| Objectives | 3 | State specific objectives, including any prespecified hypotheses. | 3,4 |
| **Methods** | | |  |
| Study design | 4 | Present key elements of study design early in the paper. | 4,5 |
| Setting | 5 | Describe the setting, locations, and relevant dates, including periods of recruitment, exposure, follow-up, and data collection. | 4,5 |
| Participants | 6 | (*a*) Give the eligibility criteria, and the sources and methods of selection of participants. Describe methods of follow-up. | 4,5 |
|  |  | (*b*) For matched studies, give matching criteria and number of exposed and unexposed. | - |
| Variables | 7 | Clearly define all outcomes, exposures, predictors, potential confounders, and effect modifiers. Give diagnostic criteria, if applicable. | 5,6 |
| Data sources/ measurement | 8* | For each variable of interest, give sources of data and details of methods of assessment (measurement). Describe comparability of assessment methods if there is more than one group. | 5,6 |
| Bias | 9 | Describe any efforts to address potential sources of bias. | - |
| Study size | 10 | Explain how the study size was arrived at. | 7,8 |
| Quantitative variables | 11 | Explain how quantitative variables were handled in the analyses. If applicable, describe which groupings were chosen and why. | 5,6 |
| Statistical methods | 12 | (*a*) Describe all statistical methods, including those used to control for confounding. | 7,8 |
|  |  | (*b*) Describe any methods used to examine subgroups and interactions. | 7,8 |
|  |  | (*c*) Explain how missing data were addressed. | - |
|  |  | (*d*) If applicable, explain how loss to follow-up was addressed. | - |
|  |  | (*e*) Describe any sensitivity analyses. | 7,8 |
| **Results** | | |  |
| Participants | 13* | (*a*) Report numbers of individuals at each stage of study - eg numbers potentially eligible, examined for eligibility, confirmed eligible, included in the study, completing follow-up, and analysed. | Table 1, Table 2 |
|  |  | (*b*) Give reasons for non-participation at each stage. | - |
|  |  | (*c*) Consider use of a flow diagram. | Figure 1 |
| Descriptive data | 14* | (*a*) Give characteristics of study participants (eg demographic, clinical, social) and information on exposures and potential confounders. | Table 2 |
|  |  | (*b*) Indicate number of participants with missing data for each variable of interest. | Table 1, Table 2 |
|  |  | (*c*) Summarise follow-up time (eg, average and total amount). | 6 |
| Outcome data | 15* | Report numbers of outcome events or summary measures over time. | 8, 12,13,14,15 |
| Main results | 16 | (*a*) Give unadjusted estimates and, if applicable, confounder-adjusted estimates and their precision (eg, 95% confidence interval). Make clear which confounders were adjusted for and why they were included. | 13,15 |
|  |  | (*b*) Report category boundaries when continuous variables were categorized. | 4,6,13,15 |
|  |  | (*c*) If relevant, consider translating estimates of relative risk into absolute risk for a meaningful time period. | - |
| Other analyses | 17 | Report other analyses done - eg analyses of subgroups and interactions, and sensitivity analyses. | - |
| **Discussion** | | |  |
| Key results | 18 | Summarise key results with reference to study objectives. | 16,17 |
| Limitations | 19 | Discuss limitations of the study, taking into account sources of potential bias or imprecision. Discuss both direction and magnitude of any potential bias. | 18 |
| Interpretation | 20 | Give a cautious overall interpretation of results considering objectives, limitations, multiplicity of analyses, results from similar studies, and other relevant evidence. | 17,18,19 |
| Generalisability | 21 | Discuss the generalisability (external validity) of the study results. | 19 |
